# The SOX2-Interactome in Brain Cancer Cells Identifies the Requirement of MSI2 and USP9X for the Growth of Brain Tumor Cells

**DOI:** 10.1371/journal.pone.0062857

**Published:** 2013-05-07

**Authors:** Jesse L. Cox, Phillip J. Wilder, Joshua M. Gilmore, Erin L. Wuebben, Michael P. Washburn, Angie Rizzino

**Affiliations:** 1 Eppley Institute for Research in Cancer and Allied Diseases, University of Nebraska Medical Center, Omaha, Nebraska, United States of America; 2 Stowers Institute for Medical Research, Kansas City, Missouri, United States of America; 3 Department of Pathology and Laboratory Medicine, The University of Kansas Medical Center, Kansas City, Kansas, United States of America; University of Pittsburgh, United States of America

## Abstract

Medulloblastomas and glioblastomas, the most common primary brain tumors in children and adults, respectively, are extremely difficult to treat. Efforts to identify novel proteins essential for the growth of these tumors may help to further our understanding of the biology of these tumors, as well as, identify targets for future therapies. The recent identification of multiple transcription factor-centric protein interaction landscapes in embryonic stem cells has identified numerous understudied proteins that are essential for the self-renewal of these stem cells. To identify novel proteins essential for the fate of brain tumor cells, we examined the protein interaction network of the transcription factor, SOX2, in medulloblastoma cells. For this purpose, Multidimensional Protein Identification Technology (MudPIT) identified >280 SOX2-associated proteins in the medulloblastoma cell line DAOY. To begin to understand the roles of SOX2-associated proteins in brain cancer, we focused on two SOX2-associated proteins, Musashi 2 (MSI2) and Ubiquitin Specific Protease 9x (USP9X). Recent studies have implicated MSI2, a putative RNA binding protein, and USP9X, a deubiquitinating enzyme, in several cancers, but not brain tumors. We demonstrate that knockdown of MSI2 significantly reduces the growth of DAOY cells as well as U87 and U118 glioblastoma cells. We also demonstrate that the knockdown of USP9X in DAOY, U87 and U118 brain tumor cells strongly reduces their growth. Together, our studies identify a large set of SOX2-associated proteins in DAOY medulloblastoma cells and identify two proteins, MSI2 and USP9X, that warrant further investigation to determine whether they are potential therapeutic targets for brain cancer.

## Introduction

Glioblastomas (GB) and medulloblastomas (MB) are highly debilitating diseases that are very difficult to treat. Despite improved therapeutic regimes, patients diagnosed with GB, the most common primary adult brain tumor, have a median survival of 10–14 months [1]. Treatment of patients with MB, the most common pediatric brain cancer, poses an additional problem. Current therapies for MB cause dramatic impairment of cognitive function and predispose patients to future treatment-associated neoplasms [2]. Hence, there is a pressing need to identify novel proteins and signaling pathways that can serve as new targets for improved treatment of GB and MB. Relevant to the work described in this study, elevated levels of the transcription factor SOX2, which plays critical roles in the development of the nervous system, have been shown to correlate with poor clinical outcome for brain tumor patients [3]. The critical role of SOX2 in brain tumors is supported by the finding that knockdown of SOX2 by RNA interference reduces the *in vitro* and *in vivo* growth of GB cells [4]. Moreover, SOX2 is expressed in MB cells [3] and, recently, we have determined that the knockdown of SOX2 in DAOY MB cells reduces their proliferation (Cox and Rizzino, unpublished results).

During the past 10 years, considerable effort has been devoted to understanding the mechanisms by which essential transcription factors mediate their effects. More recently, significant strides have been made toward mapping protein-protein interaction landscapes of essential transcription factors in a number of cellular systems. For example, extensive progress has been made in determining the proteome of transcription factors, in particular Sox2, Oct4 and Nanog, necessary for maintaining the self-renewal and pluripotency of embryonic stem cells (ESC) [5]–[11]. The integration of interactomes for Sox2, Oct4 and Nanog, argues that these pluripotency associated transcription factors are part of a highly integrated protein-protein interaction landscape, which includes many other transcription factors, chromatin remodeling machinery, DNA repair machinery and RNA binding proteins [9], [11]–[13]. Furthermore, unbiased proteomic screens to identify proteins that associate with Sox2 in mouse ESC have proven to be a powerful approach for identifying under-studied proteins, such as Banf1 and Musashi2 (MSI2), that significantly influence the fate of ESC [11]–[15]. Given that SOX2 associates with a diverse array of essential proteins, it is likely that proteomic analysis of the SOX2-interactome in brain tumor cells could help identify additional proteins that influence the growth of these tumors.

To improve our understanding of brain tumors, the work reported in this study set out to address two questions. What is the composition of the SOX2-interactome in the MB tumor cell line DAOY? Can the proteomic screen of SOX2-associated proteins help identify additional proteins that are required by brain tumor cells? We report that SOX2 associates with >280 proteins in DAOY cells. In addition, we demonstrate that two SOX2-associated proteins, MSI2 and Ubiquitin Specific Peptidase 9x (USP9X), which have been recently implicated in the growth of other cancers [16]–[21], are required to support the growth and survival of DAOY cells and two GB tumor cell lines, U87 and U118.

## Experimental Procedures

### Cell Culture

DAOY (HTB-186, ATCC, Manassas, VA), i-SOX2-DAOY, U87 (HTB-14, ATCC), U118 (HTB-15, ATCC) and HEK293T (CRL-11268, ATCC) cells were cultured, as described previously [22]. Lentiviral particles were produced, and cells were infected, as described previously [15]. Cell growth was examined using the MTT assay, as described previously [15] using cells replated 3–4 days after lentiviral infection.

### Plasmid Production

FUW-tetO-SOX2 (20724) was obtained from Addgene (Cambridge, MA). pLVX-Tet-On® Advanced vector was obtained from Clontech (632162, Mountain View, CA). Vectors to produce shRNA lentiviruses for MSI2 (RMM4534-NM_011443) and USP9X (RHS4533-NM_001039590) knockdowns were obtained from Open Biosystems (Huntsville, AL). A previously validated non-specific shRNA (Scrambled) was used as a negative control in knockdown experiments [23]. shRNA sequences are provided in supplemental Table S4.

To engineer pLVX-tetO-(fs)SOX2, Strep and Flag tags were sequentially added to the N-terminus of SOX2 by two rounds of PCR. The first PCR round used FUW-tetO-SOX2 as a template, the upstream primer: CAAGAAGCTTGCC*AACTGGAGCCACCCACAATTCGAGAAG*GGCGGA**ATGTATAACATGATGGAGACGGAGCTGAAG** (underlined: HindIII, italicized: Strep-epitope, bold: SOX2 CDS, bold underlined: silent mutation to destroy an endogenous BsrGI site) and the downstream primer: AGGACTCGAGCGCGGGACCACACCATGAAGG (underlined: XhoI). This fragment was digested with HindIII and XhoI and ligated into corresponding sites of Bluescript KS+ (Sratagene, Santa Clara, CA). The second round of PCR, using the intermediate plasmid as template, was run with the following upstream primer: AAGGTCTAGATGTAC***ATGGACTACAAGGACGACGATGACAAG***GGGTCGGCCGCC*AACTGGAGCCACCCACAATTCGAGAAG* (underlined: XbaI, gray: BsrGI, bold and italicized: Flag-epitope, italicized: Strep-epitope) and the downstream primer containing the XhoI site described above. This PCR product was then digested with XbaI and XhoI and ligated into corresponding sites of Bluescript. The Flag-Strep tagged 5′ end of SOX2 was isolated from this intermediate vector and transferred into the FUW-tetO-SOX2 vector using BsrGI and an internal RsrII site, to create FUW-tetO-(fs)SOX2. (fs)SOX2 was isolated from FUW-tetO-(fs)SOX2 using EcoRI and ligated into pLVX-Tight-Puro (632162, Clontech), to create pLVX-tetO-(fs)SOX2. Orientation was verified by sequencing.

### i-SOX2-DAOY Cell Engineering

DAOY cells were seeded at clonal density and infected with pLVX-Tet-On® Advanced lentivirus to produce rtTA-DAOY. Cells were refed fresh medium 2–3 times per week. Eight days after seeding at clonal density, individual colonies were isolated, and two days later, isolated colonies were exposed to G418 (300 µg/mL). rtTA-DAOY clones were expanded for ∼2 weeks under G418 selection. rtTA-DAOY cells were seeded at clonal density and infected with pLVX-tetO-(fs)SOX2. Cells were refed fresh medium 2–3 times per week. Eight days after seeding, cells were exposed to puromycin (5 µg/mL) for 72 hours, after which 6 clones were isolated. Each clone was cultured in medium supplemented with doxycycline (0.1 µg/mL) for 24 hours, and nuclear proteins were isolated. Inducible expression of (fs)SOX2 was verified by western blot analysis (data not shown). A single clone, referred to as i-SOX2-DAOY, was used for proteomic analysis.

### Protein Isolation and Immunoprecipitation

Dounce homogenization was used to isolate nuclear proteins for MudPIT analysis as described previously [24]. For immunoprecipitation, nuclear extracts were loaded onto M2-beads (Sigma-Aldrich, St. Louis, MO) and washed, and protein complexes were eluted using 3X Flag-peptide (Sigma-Aldrich), as described previously [9]. Differential pulldown between the induced (+Dox) and uninduced (−Dox) samples was verified by silver staining [9].

### MudPIT Identification of SOX2-associated Proteins

Multidimensional Protein Identification Technology (MudPIT) analysis was performed as described previously [9]. The MS/MS datasets were examined using SEQUEST and the *Homo sapiens* protein database (NCBI, 2010-11-22 release). Distributed Normalized Spectral Abundance Factors (dNSAF) were calculated for each detected protein, as described elsewhere [25]. Three independent experiments and statistical analysis were performed as described previously [9]. The spectral false discovery rate was determined as described previously [9], [26].

### Western Blot Analysis

Western blot analysis was performed as described previously [9]. Nuclear and cytoplasmic protein extracts were prepared using NE-PER kits (Thermo-Scientific, Rockford, IL) was used according to the manufacturer’s protocol to isolate proteins for western blot analysis, as described previously [9]. Relative levels of detected proteins were determined as described previously [11]. Primary antibodies used were: α-MSI2 (ab83236, Abcam, Cambridge, MA), α-USP9X (ab56461, Abcam), α-USP7 (A300-033A, Bethyl Laboratories, Montgomery, TX), α-Flag (F3165, Sigma-Aldrich), α-GAPDH (G8795, Sigma-Aldrich), α-NUMB (ab4147, Abcam), α-SOX2 (abl5830, Abcam), α-MCL1 (sc-819, Santa Cruz Biotechnology, Santa Cruz, CA), α-β-Catenin (9562, Cell Signaling Technology, Danvers, MA).

### RNA Analysis

RNA isolation and cDNA synthesis performed as described previously [9]. Gene expression of MSI2 and USP9X in DAOY, U87, or U118 cells treated with targeting or scrambled shRNA were analyzed by SYBR Green (SuperArrayBioscience Corporation, Federick, MD) quantitative Real-Time polymerase chain reaction (RT-qPCR) [9]. Primers used for the PCR step in the analysis of MSI2 RNA were h-MSI2-F (AAGTATTAGGTCAGCCCCAC) and h-MSI2-R (TTCTCAAAAGTGACAAAGCC). Primers used for the PCR step in the analysis of USP9X RNA were h-USP9X-F (CAGATGACCAAGATGCTCC) and h-USP9X-R (GGGGATACTTCTTCACTGCC). Primers for GAPDH control expression have been described previously [15].

## Results

### Engineering of i-SOX2-DAOY Cells and MudPIT Analysis of SOX2-associated Proteins

**Figure 1:**
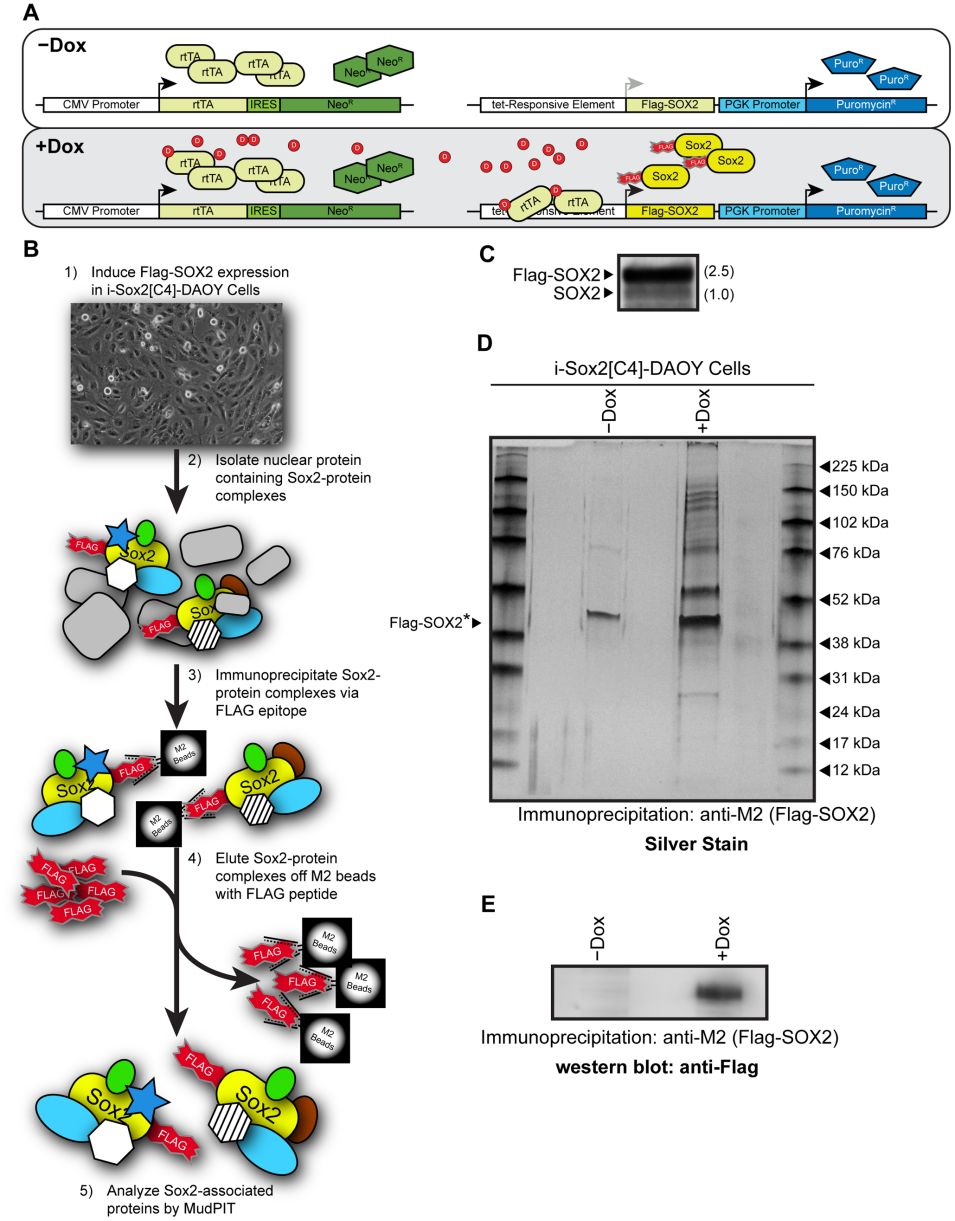
Engineering of iSOX2-DAOY medulloblastoma cells to identify SOX2-associated proteins. (A) Schematic diagram of the lentiviral system used to introduce a Dox-inducible, epitope tagged SOX2 into DAOY MB cells. Two lentiviral vectors were used to introduce constitutively expressed reverse-tet transactivator (rtTA) and inducible (fs)SOX2 [labeled: Flag-SOX2]. (B) Protocol used to isolate SOX2-protein complexes from DAOY MB cells for downstream MudPIT analysis. (C) Western blot analysis probing for SOX2 with a Sox2 antibody. The level of (fs)SOX2 was compared to the level of endogenous SOX2, which was set to 1. (D) Silver stain demonstrating enrichment of proteins following the induction of (fs)SOX2 with Dox and immunoprecipitation with M2-beads. The prominent band observed at ∼45 kDa is a common contaminant when M2-beads are used for immunoprecipitation. *The estimated position of (fs)SOX2 is indicated by an arrowhead. (E) Western blot analysis probing for Flag-SOX2 to verify immunoprecipitation using M2-beads.

To help identify novel proteins essential for growth of MB cells, we examined the SOX2-interactome in DAOY MB cells. Importantly, transcription factors, such as SOX2, do not work in isolation, but function as part of large protein complexes. In this regard, previous studies conducted with ESC undergoing differentiation have shown that Sox2 is present in multiple large molecular weight protein complexes, some exceeding 880 kDa [27]. To examine the SOX2-interactome, DAOY cells were engineered for the inducible expression of epitope-tagged SOX2 (i-SOX2-DAOY), because antibodies directed against SOX2 are not suitable for highly specific and sensitive isolation of SOX2-protein complexes. This enabled us to consistently isolate SOX2 and its associated proteins using M2-beads. To engineer i-SOX2-DAOY, DAOY cells were sequentially infected with a lentivirus to introduce a constitutively expressed reverse-tet transactivator and a second lentiviral vector that expresses a Flag-Strep dual epitope tagged form of SOX2 [(fs)SOX2] under the control of a Dox-inducible promoter (Fig. 1A).

To isolate (fs)SOX2 and its associated protein complexes from i-SOX2-DAOY cells, (fs)SOX2 expression was induced for 24 hours (0.1 µg/mL Dox), and (fs)SOX2 protein complexes were isolated from nuclear protein extracts by immunoprecipitation using M2-beads (Fig. 1B). As a control, nuclear extracts were prepared from i-SOX2-DAOY cells without exposure to Dox (uninduced samples). Following induction with Dox, the level of (fs)SOX2, which migrates slightly slower than SOX2, was found to be ∼2.5-fold higher than that of endogenous SOX2 (Fig. 1C). However, if expression of exogenous SOX2 reduces the expression of endogenous SOX2 as it does in ESC [11], the total levels of SOX2 in the Dox-treated cells was less than 2.5-fold higher than that in the untreated DAOY cells. Importantly, silver stain analysis of immunoprecipitated eluates demonstrated a significant enrichment of proteins isolated when (fs)SOX2 was induced (Fig. 1D). As expected, (fs)SOX2 could be readily detected by western blot analysis in the induced, but not in the uninduced eluates (Fig. 1E). The prominent band observed at ∼45 kDa (Fig. 1D) is a common contaminant when M2-beads are used for immunoprecipitation [9], [11].

**Figure 2:**
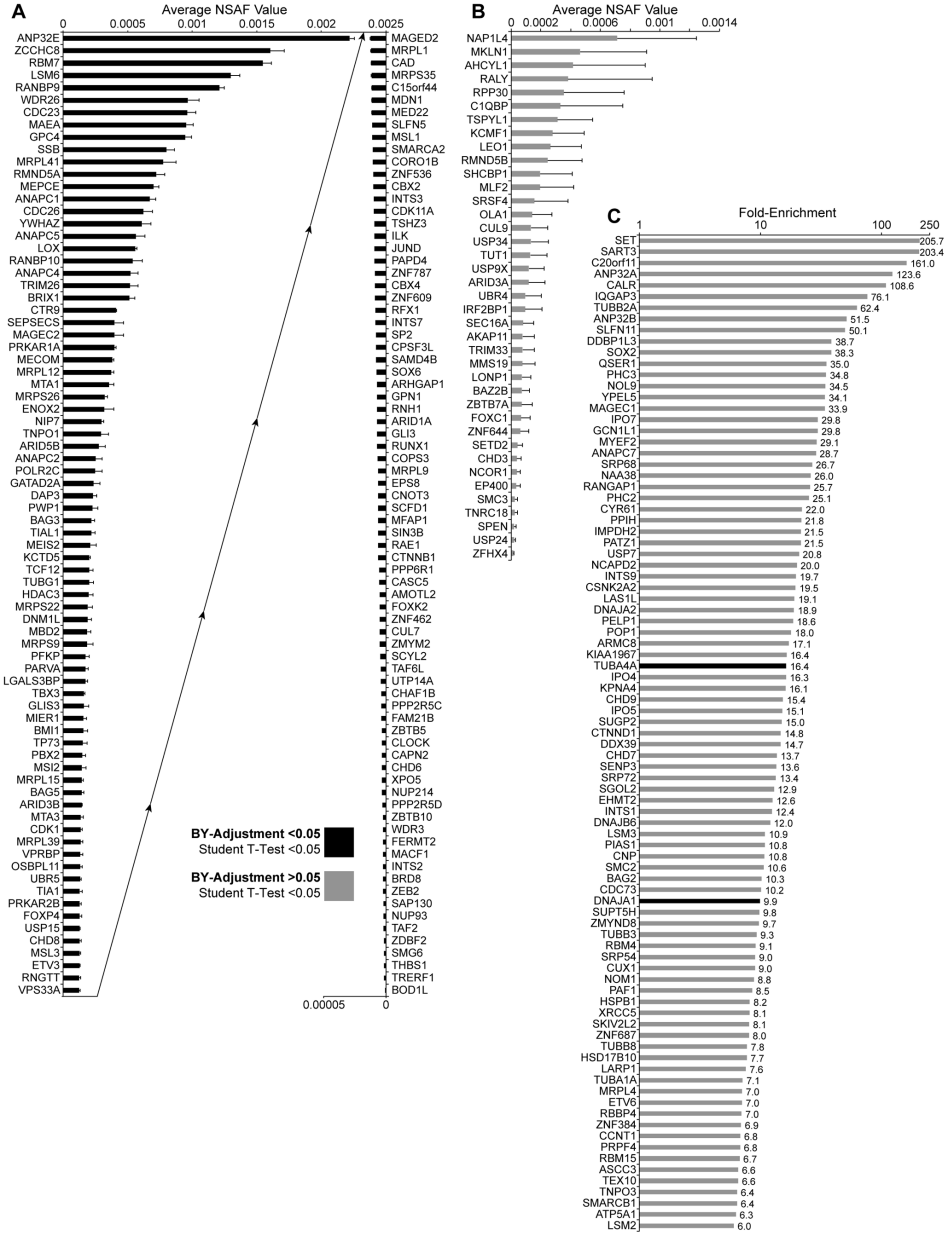
SOX2-associated proteins identified in DAOY MB tumor cells by MudPIT analysis. (A) Proteins that were identified in all three induced M2-bead eluates, but not identified in uninduced samples, as SOX2-associated proteins by MudPIT analysis, which were statistically significant according to the BY-adjusted method (p<0.05). NSAF values of the three replicates are averaged and the error bars represent standard deviation. The plot is split in two; the ‘Average NSAF Values’ of the left half ascend left to right, and the ‘Average NSAF Values’ of the right half ascend right to left. (B) Proteins identified in 3 of 3, Dox-induced, but not in uninduced, DAOY MudPIT replicates that were statistically significant according to the student’s t-test (p<0.05), but were not significant according to the BY-adjustment (p>0.05). (C) Proteins that were identified in all three induced but at least one uninduced MudPIT sample, are plotted according to fold enrichment (NSAF values) in Dox-induced samples compared to uninduced. Only proteins with enrichment values >6-fold were included. Proteins depicted with a black bar had enrichment values that were statistically significant according to the BY-adjustment (p<0.05). Proteins depicted with gray bars were statistically significant according to the student’s t-test (p<0.05), but not significant according to the BY-adjustment (p>0.05).

To identify proteins that associate with SOX2 in i-SOX2-DAOY cells, SOX2-protein complexes from uninduced and induced i-SOX2-DAOY cells were subjected to MudPIT analysis. To help minimize experimental variation and for statistical analysis, MudPIT analysis was performed on three independent pairs of samples. The spectral false discovery rate of our proteomic screens were 0.27%±0.16 and 0.37%±0.08 for uninduced and induced samples, respectively, as determined by the method of Elias and colleagues [26]. Proteins identified as SOX2-associated proteins were grouped into three major categories in an effort to minimize the exclusion of bona-fide SOX2-associated proteins: 1) proteins identified only in our induced samples in three MudPIT analyses with the more stringent BY-adjusted significance p<0.05 (Fig. 2A, supplemental Table S1); 2) proteins identified only in induced samples in three MudPIT analyses without BY-adjusted significance (p>0.05), but with t-test significance (p<0.05) (Fig. 2B, supplemental Table S2); and 3) proteins that were enriched in the induced sample more than 6-fold (Fig. 2C, supplemental Table S3). Category 1 and category 2 totaled 156 and 39 proteins, respectively. All proteins in category 1 met the statistical requirement used for category 2. Together, with category 3, which included 88 proteins, a total of 283 SOX2-associated proteins were identified when all three categories were combined.

**Figure 3:**
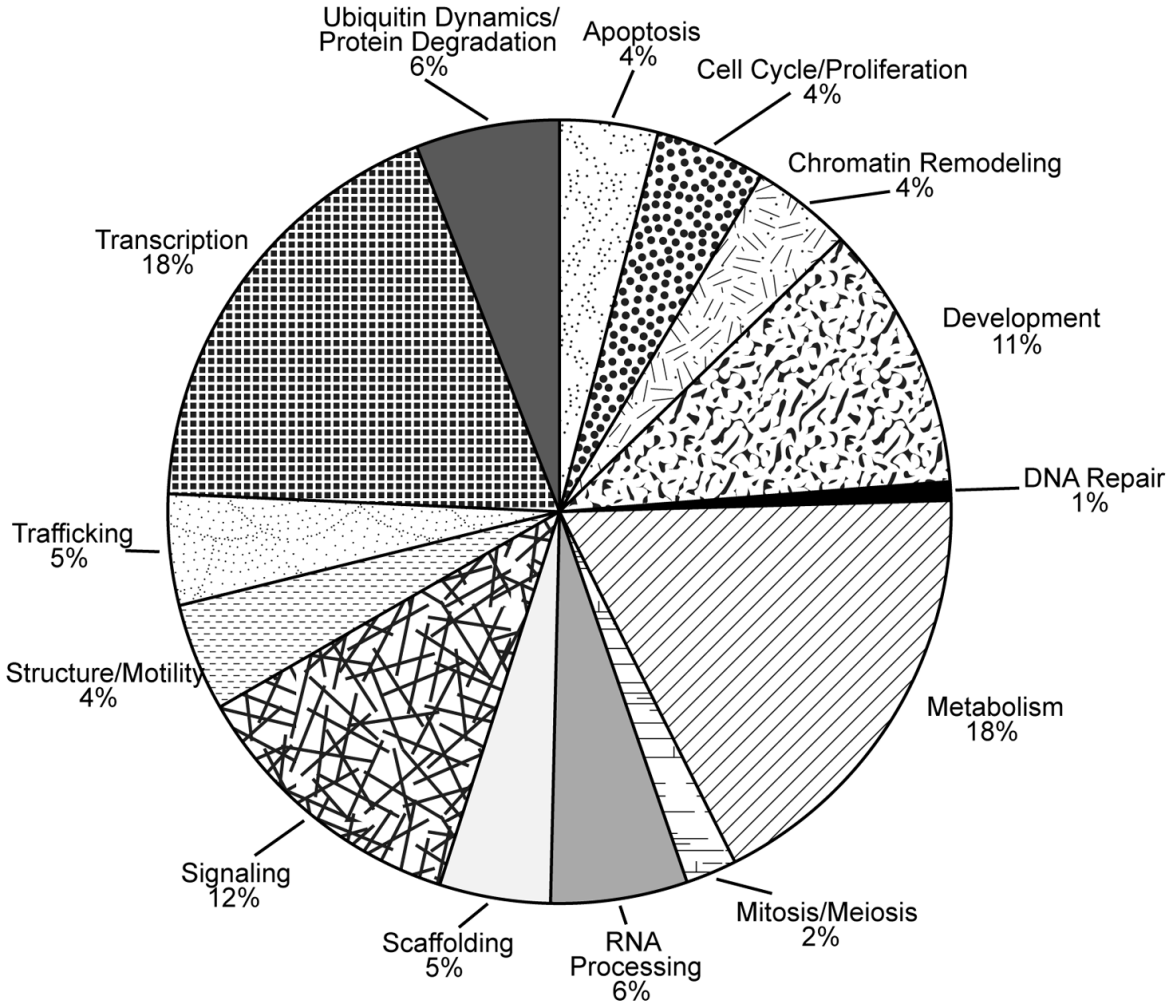
Gene ontology analysis of SOX2-associated proteins identified in MB tumor cells. Gene ontology analysis of SOX2-associated proteins identified in DAOY MB cells was conducted using the Database for Annotation, Visualization and Integrated Discovery (DAVID). Specific ontology descriptions for SOX2-associated proteins were grouped into 14 broad categories.

To better understand the biological functions of SOX2-associated proteins, we performed gene ontology classification with Database for Annotation Visualization and Integrated Discovery (DAVID). Given the nuclear location and function of SOX2, it is not surprising that transcription is one of the largest functional categories (Fig. 3). However, SOX2-associated proteins have been linked to many other cellular processes. This is similar to the finding that Sox2-associated proteins in mouse ESC and in mouse ESC undergoing differentiation are involved in a diverse array of functions [9], [11]. In these cellular contexts, a similar percentage of SOX2-associated proteins participated in the categories of RNA processing (6% to 9%) and development (10% to 11%). However, there were several notable differences. The percentage of SOX2-associated proteins that fall under the DNA repair category represented 11% in ESC undergoing differentiation [9], but only 1% in DAOY cells. The classification for each SOX2-associated protein is provided in supplemental Table S5.

MudPIT is a highly sensitive method that is able to detect proteins in samples that are not readily detected by western blot analysis. For this reason, we used a more traditional biochemical approach and selected proteins with modest NSAF values to validate our identification of SOX2-associated proteins. Specifically, MSI2, a protein found only in our induced samples (Fig. 2A), and USP7, enriched ∼20-fold (Fig. 2C), were confirmed to associate with SOX2 through co-immunoprecipitation using nuclear extracts prepared from DAOY cells (supplemental Fig. S1A). We also determined that SOX2 is able to associate with MSI2 and USP7 in other cellular contexts. In this regard, MSI2 and USP7 were identified as SOX2-associated proteins in a single proteomic screen of SOX2-associated proteins in U87 GB cells (Wilder and Rizzino, unpublished results). Additionally, we determined that ectopically expressed Flag-SOX2 associates with endogenous USP7 found in HEK293T cells (supplemental Fig. S1B). We also confirmed that ectopically expressed Flag-MSI2 co-immunoprecipitates ectopically expressed SOX2 in HEK293T cells (supplemental Fig. S1C).

### Integration of SOX2-protein Interactomes Reveals a Number of Common Associating Proteins

In addition to the SOX2 proteomic screen conducted in DAOY cells, we have recently conducted MudPIT analysis to identify SOX2-associated proteins in several other cellular contexts, including undifferentiated ESC [11] and ESC undergoing differentiation [9]. Importantly, the three SOX2-interactomes identified were determined using the same methods for protein isolation and proteomic analysis, and a similar spectral false discovery rate (<0.4%) was determined in each independent context [9], [11]. In each case, Flag-epitope tagged SOX2 and its associated proteins were isolated using M2-beads from nuclear extracts prepared by Dounce homogenization, followed by MudPIT analysis using the same proteomic platform. Therefore, we compared SOX2-interactomes identified in each of these cellular contexts (supplemental Fig. 2, supplemental Table S6), because proteins that associate with SOX2 in multiple cellular contexts are also likely to play essential roles in the behavior of brain tumor cells. In this regard, human MB cells and mouse ESC had several SOX2-associated proteins in common, despite dramatic differences in cellular context. Of 283 proteins identified in the SOX2-interactome in DAOY MB cells, 19 associate with SOX2 in at least one other cellular context, including MSI2 and USP9X, and two associate with SOX2 in all three cellular contexts (supplemental Fig. S4, supplemental Table S6). Recently, we have shown that Msi2 is required for the self-renewal and pluripotency of mouse ESC [14] and, more recently, we have determined that knockdown of Usp9x in mouse ESC substantially increases the differentiation of ESC (unpublished results). Furthermore, MSI2 and USP9X have been implicated in other cancers [16]–[21], but their roles in brain tumors have not been examined. Therefore, we expanded our analysis of SOX2-associated proteins by determining whether decreasing MSI2 and USP9X expression influences the behavior of brain tumor cells.

### Musashi-2 is Necessary for the Proliferation of Brain Cancer Cells

**Figure 4:**
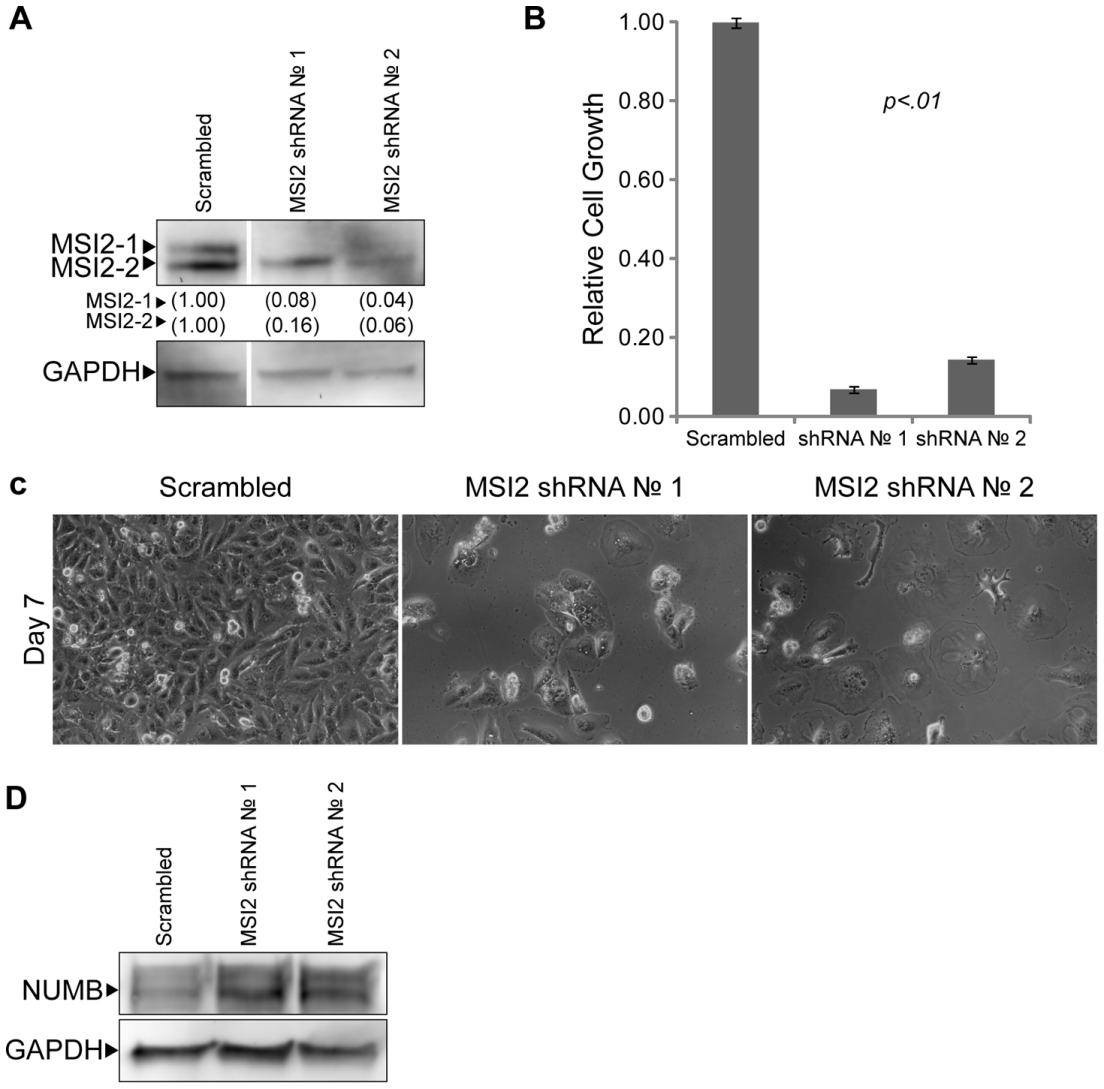
Knockdown of MSI2 in DAOY MB cells. (A) Western blot analysis of MSI2 levels in DAOY whole cell extracts 96 hours after infection with Scrambled or MSI2 shRNA lentiviruses. Two isoforms were detected: isoform 1 (MSI2-1) and isoform 2 (MSI2-2). GAPDH was probed as a loading control. MSI2 levels are quantified, with levels found in the Scrambled control set to 1.00. (B) Cell growth was examined in triplicate by MTT assay 6 days after being plated at 10^4^ cells per well of a 12-well plate. The data shown are averages relative to the Scramble control. Error bars represent standard deviation. P values were determined by student t-test and found to be <.01 for both MSI2 shRNA 1 and 2. (C) Photomicrographs of DAOY MB cells following infection with either non-specific (Scrambled) or MSI2 targeting (No. 1, No. 2) shRNA lentiviruses. Cells were infected on Day 0, selected using medium supplemented with puromycin on Day 1 and refed fresh medium on Day 2. Cells were photographed on day 7 after infection. (D) Western blot analysis of NUMB in DAOY MB extracts used in Figure 4A.

Previous reports demonstrated that MSI2 is essential for the progression of CML [16]–[18], and the knockdown of another family member, Musashi-1 (MSI1), disrupts the viability of DAOY MB cells and GB cells [28], [29]. To determine whether MSI2 is necessary for the proliferation of DAOY MB cells, shRNA constructs were used to knock down endogenous MSI2. Specifically, lentiviruses that constitutively express shRNA against MSI2 were used to infect DAOY MB cells. Two independent shRNA constructs were used to knockdown MSI2, and a previously characterized non-specific shRNA (Scrambled) was used as a control [15], [23]. Following selection of the infected cells with puromycin, western blot analysis demonstrated that MSI2 isoforms 1 and 2 were substantially knocked down (Fig. 4A). This reduction in MSI2 was verified at the RNA levels by RT-qPCR (Supplemental Fig. S3A). In addition, when compared to the growth of DAOY cells infected with the Scrambled shRNA lentiviral vectors, we observed a large reduction in growth when the cells were infected with MSI2 shRNA lentiviral vectors (Fig. 4B). Moreover, photomicrographs taken 7 days after infection demonstrated that cells in which MSI2 had been knocked down were flatter and larger, reminiscent of post-mitotic cells, when compared to the Scrambled control (Fig. 4C).

Currently, relatively little is known about the roles of MSI2, but in mouse model of leukemia it is believed to down-regulate the protein Numb [16], which has been shown to regulate both Notch and Wnt signaling [30], [31]. Therefore, we examined whether knockdown of MSI2 in DAOY cells influences the expression of NUMB. We determined that knockdown of MSI2 with shRNA lentiviral vectors #1 and #2 caused an increase in the protein levels of NUMB (Fig. 4D). Thus, knockdown of MSI2 causes both a large reduction in the growth of DAOY MB tumor cells and increases the expression of NUMB.

**Figure 5:**
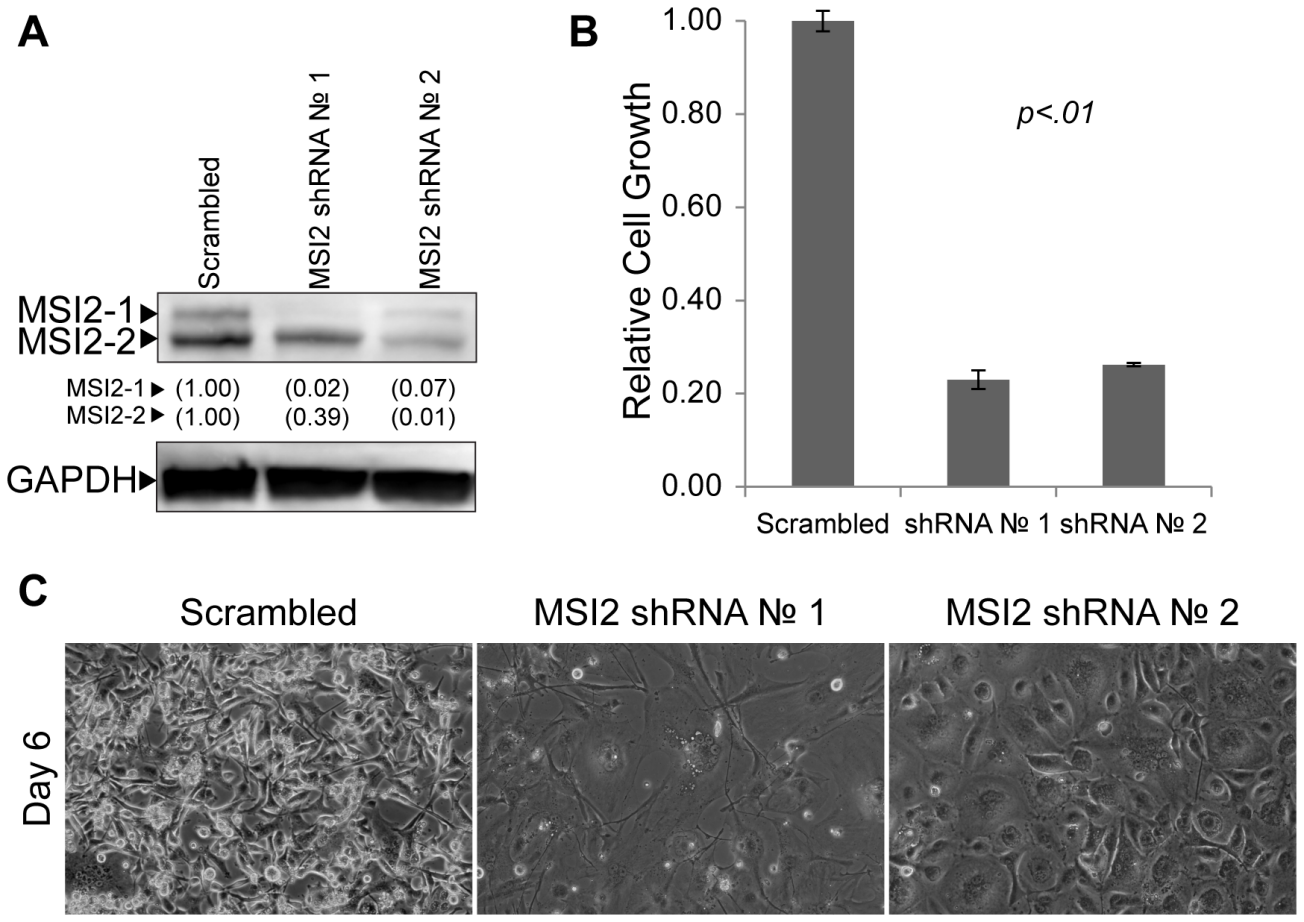
Knockdown of MSI2 in U87 GB cells. (A) Western blot analysis of MSI2 levels in U87 nuclear extracts 96 hours after infection with Scrambled or MSI2 shRNA lentiviruses. MSI2 levels are quantified, with levels found in the Scrambled control set to 1.00. (B) Cell growth was examined in triplicate by MTT assay 5 days after being plated at 1.5×10^4^ cells per well of a 12-well plate. The data shown are averages relative to the Scramble control. Error bars represent standard deviation. P values were determined by student t-test and found to be <.01 for both MSI2 shRNA 1 and 2. (C) Photomicrographs of U87 GB cells following infection with either non-specific (Scrambled) or MSI2 targeting (No. 1, No. 2) shRNA lentiviruses. Cells were infected on Day 0, selected using medium supplemented with puromycin on Day 1 and refed fresh medium on Day 3. Cells were photographed on day 6 after infection.

We also examined the consequences of knocking down MSI2 in GB tumor cells, because MSI2 was also identified as a SOX2-associated protein in U87 GB cells (Wilder and Rizzino, unpublished results). For this purpose, we initially infected U87 GB tumor cells with the same MSI2 shRNA lentiviral vectors described earlier. Again, a scrambled shRNA was used as a control. Three days after infection with the lentiviral vectors, western blot analysis determined that MSI2 isoform 1 and isoform 2 were both substantially reduced (Fig. 5A) and the reduction in MSI2 was verified at the RNA levels by RT-qPCR (supplemental Fig. S3B). As in the case of DAOY cells, U87 GB cells infected with MSI2 shRNA constructs exhibited a marked decrease in cell proliferation (Fig. 5B) and a significant increase in cell size (Fig. 5C). To extend these findings, U118 GB cells were infected with MSI2 shRNA lentiviruses. Similar to DAOY and U87 cells, knockdown of MSI2 in U118 cells resulted in a decrease in MSI2 protein and RNA, a large reduction in cell growth, and a significant increase in cell size (supplemental Fig. S4 and Fig. S3C). Taken together, our data indicate that MSI2 is required to sustain the survival of DAOY MB cells, and the proliferative capacity of U87 and U118 GB cells.

### Knockdown of USP9X Reduces the Viability of Brain Cancer Cells

**Figure 6:**
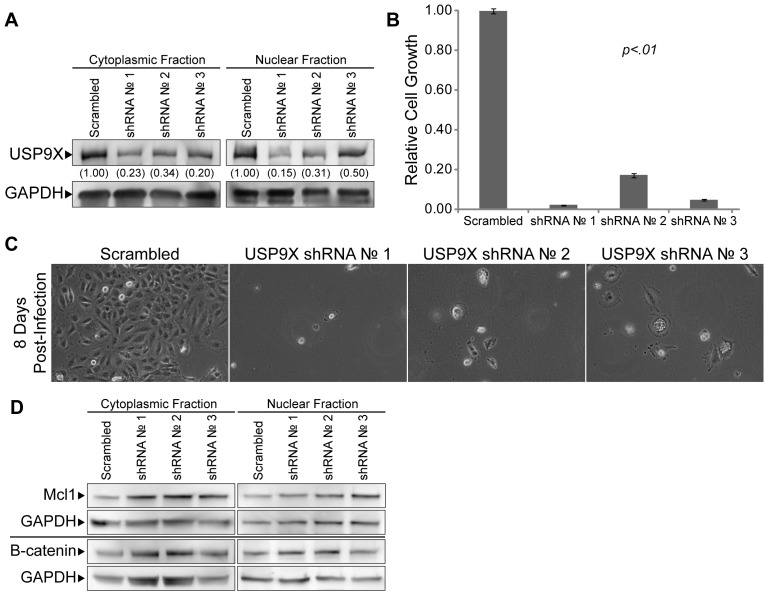
Knockdown of USP9X in DAOY MB. (A) Western blot analysis to verify the knockdown of USP9X in DAOY MB cells following infection with lentiviruses to introduce constitutively active shRNA against USP9X transcripts. Nuclear and cytoplasmic protein fractions were prepared 4 days after infecting cells with lentiviruses. USP9X levels are quantified, and levels in the Scrambled control are set to 1.00. (B) Cell growth was examined in triplicate by MTT assay 6 days after being plated at 10^4^ cells per well of a 12-well plate. The data shown are averages relative to the Scramble control. Error bars represent standard deviation. P values were determined by student t-test and found to be <.01 for both USP9X shRNA 1, 2 and 3. (C) Photomicrographs of DAOY MB cells following knockdown of USP9X using lentiviral delivered shRNA constructs against USP9X transcripts. On day 0, cells were infected with USP9X shRNA lentiviruses. Beginning on day 1, infected cells were selected using puromycin for 48 hours. On day 4, cells were passaged into fresh culture flasks (5×10^5^ cells per flask) and photographed on day 8 post-infection. (D) Western blot analysis of Mcl1, and β-catenin – targets of USP9X – in DAOY MB extracts used in Figure 6A.

We also examined whether USP9X is required for the survival of brain cancer cells. For this purpose, we utilized shRNA mediated knockdown of USP9X. More specifically, three independent lentiviruses were used to deliver constitutively active shRNA constructs against USP9X transcripts in DAOY MB cells. As with our MSI2-knockdown studies, Scrambled shRNA was used as a control. Knockdown of USP9X was confirmed by western blot analysis of both nuclear and cytoplasmic extracts (Fig. 6A) and verified at the RNA levels by RT-qPCR (supplemental Fig. S5A). Although there were minimal effects of knocking down USP9X during the first three days of culture, we observed a large reduction in the number of cells that could reattach (data not shown). Moreover, the few cells from the USP9X knockdown population that reattached after subculture exhibited little if any proliferation (Fig. 6, B and C). In addition, we examined whether the knockdown of USP9X affects the expression of several of its known targets in DAOY cells. USP9X has been reported to deubiquitinate a large number of proteins, including MCL1 and β-catenin [19], [32]–[35]. However, knockdown of USP9X did not appear to alter the expression of MCL1 or β-catenin in the nuclear or cytoplasmic compartments (Fig. 6D).

**Figure 7:**
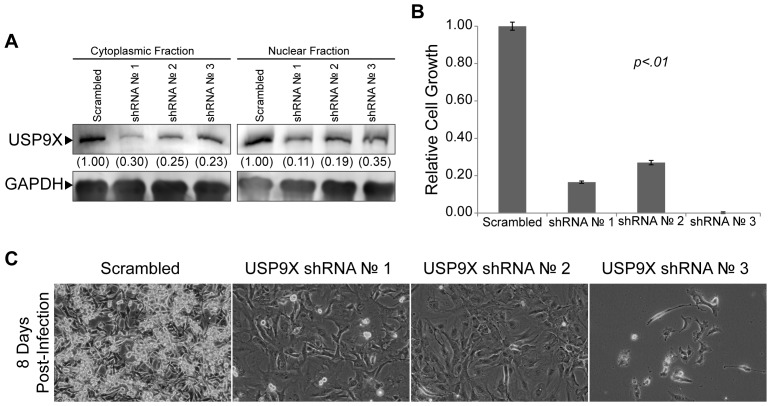
Knockdown of USP9X in U87 GB cells. (A) Western blot analysis to verify the knockdown of USP9X in U87 GB cells following infection with lentiviruses to introduce constitutively active shRNA against USP9X transcripts. Nuclear and cytoplasmic protein fractions were prepared 4 days after infecting cells with lentiviruses. USP9X levels are quantified, and levels in the Scrambled control are set to 1.00. (B) Cell growth was examined in triplicate by MTT assay 5 days after being plated at 1.5×10^4^ cells per well of a 12-well plate. The data shown are averages relative to the Scramble control. Error bars represent standard deviation. P values were determined by student t-test and found to be <.01 for both USP9X shRNA 1, 2 and 3. (C) Photomicrographs of U87 GB cells following knockdown of USP9X using lentiviral delivered shRNA constructs against USP9X transcripts. On day 0, cells were infected with USP9X shRNA lentiviruses. Beginning on day 1, infected cells were selected using puromycin for 48 hours. On day 4, cells were passaged into fresh culture flasks (5×10^5^ cells per flask) and photographed on day 8 post-infection.

To determine whether knockdown of USP9X can perturb other brain tumor types, U87 and U118 GB tumors cells were also transduced with lentiviruses to knockdown USP9X. As in the case of DAOY cells, shRNA lentiviral vectors directed against USP9X caused a significant reduction in USP9X protein and RNA in both U87 GB cells (Fig. 7A, supplemental Fig. S5B) and U118 GB cells (supplemental Fig. S6 and supplemental Fig. S5C). Moreover, similar to DAOY cells, there were minimal effects during the first 3 days after infection with the USP9X shRNA lentiviral vectors. However, the ability of the U87 cells and U118 cells to reattach after being subcultured was reduced (data not shown), which contributed to the large reduction in U87 and U118 cell number observed in our cultures infected with USP9X shRNA lentiviral vectors (Fig. 7B, supplemental Fig. S6). Interestingly, the morphology of U87 and U118 GB cells changed significantly following the knockdown of USP9X. Similar to the results following MSI2 knockdown in DAOY cells, USP9X knockdown caused notable increases in cell size (Fig. 7C, supplemental Fig. S6). Taken together, our studies demonstrate that knockdown of USP9X drastically alters the growth characteristics of brain cancer cells.

## Discussion

In this study, we performed a proteomic screen using MudPIT to identify the SOX2-interactome in DAOY MB cells. We identified >280 SOX2-associated proteins in DAOY cells. Moreover, we determined that two of the SOX2-associated proteins, which had not been studied previously in brain tumor cells, are required for the proliferation of DAOY cells as well as two human GB cell lines. Importantly, the findings reported here indicate that our strategy of comparing the SOX2-interactomes in multiple cellular contexts is a useful approach for identifying understudied proteins that play key roles in cellular physiology and interconnect critical biological networks.

Although SOX2 associates with a large number of nuclear proteins in DAOY cells, it is unlikely that SOX2 physically interacts directly with many of these proteins. In this regard, previous studies have shown that Sox2 is present in high molecular weight complexes in ESC during the initial stages of differentiation [27]. Determining which proteins interact directly with SOX2 will require the isolation and extensive characterization of SOX2-protein complexes. Furthermore, our studies do not directly address whether the association of SOX2 with specific proteins is essential for their action or activity. Such an undertaking will not only require characterization of specific SOX2-protein complexes, but also mapping the proteins domains of each protein needed for the proper assembly of these protein complexes. Only then can one begin to generate subtle mutations that disrupt the function of these protein complexes without massively disturbing the formation of these complexes, which are likely to play multiple roles in the physiology of the cells.

Recent proteomic studies indicate that the SOX2-interactome is highly context-dependent. We have shown that the Sox2-interactome in mouse ESC changes dramatically when ESC begin to differentiate. Shortly after the induction of differentiation, there is >75% change in the Sox2-interactome [9], [11]. Even within different types of brain tumor cells, the SOX2-interactome appears to be context dependent. Comparison of the SOX2-interactome described in this study for DAOY MB tumor cells (283 SOX2-associated proteins) with the SOX2-interactome described recently for GB tumor cells (144 SOX2-associated proteins) [36] shows surprisingly little overlap. Only two proteins were identified in both SOX2-interactomes, XRCC5 and AHCTL1. It is likely that some of the differences observed in these two studies may be due to differences in the methods used to isolate SOX2-protein complexes and the mass spectrometry platforms used to characterized the proteins in these complexes. However, >60% of proteins identified as SOX2-associated proteins in the study of LN229 cells [36] were also detected our proteomic analysis of SOX2-associated proteins isolated from U87 GB cells (Wilder and Rizzino, unpublished results).

Recent studies have led to the division of MB into several subgroups, including the Shh subgroup and the WNT subgroup, based on RNA expression profiles [37]. DAOY cells resemble the Shh subgroup of MB [38], and SOX2 expression tends to be higher in this subgroup relative to other MB subgroups [39], [40]. However, as a group, SOX2-associated proteins identified in our study are not enriched in any particular subgroup. Interestingly, USP9X is expressed at a higher level in at least one of the major subgroups of MB [40]. Moreover, the SOX2-associated protein β-catenin (CTNNB1) has stabilizing mutations in ∼70% of cases belonging to the WNT-subgroup of MB [41]. Other SOX2-associated proteins identified here have also been implicated in brain cancer. The polycomb group protein BMI1, which associates with SOX2 in DAOY cells (Fig. 2) and U87 GB cells (unpublished results) has been reported to be overexpressed in MB, especially in the most aggressive subtypes [5], [42], and growth of human GB in a xenograph mouse model is retarded when BMI1 is knocked down [43]. Another SOX2-associated protein BAG3 (Bcl2-associated athanogene), which was identified in our proteomic screens conducted in DAOY and U87 cells, has been reported to be overexpressed in GB, and knockdown of BAG3 in a rat model of glioblastoma sensitizes the cells to apoptosis [44]. In yet another study, the protein levels of the SOX2-associated protein Mta1 (Fig. 2) are elevated in a glioma tumor model [45]. Additionally, knockdown of USP15 in GB, which stabilizes the type I TGF-β receptor, decreases the oncogenic capacity of patient-derived tumor cells in an orthotopic mouse model [46].

Although several SOX2-associated proteins have been implicated in brain cancer, the roles of the vast majority of SOX2-associated proteins have not been examined in brain cancer. In this report, we studied two SOX2-associated proteins, which have been implicated in supporting the growth of other cancers. Knockdown of MSI2 in MB and GB cells impairs their ability to proliferate. Currently, it is unclear why the knockdown of MSI2 reduces the growth of brain tumor cells. Recent studies suggest that the translation of NUMB mRNA, which is a known target of MSI1 [47], inversely correlates with MSI2 expression in leukemia [18]. Interestingly, we observed an increase in the level of NUMB when MSI2 was knocked down in DAOY cells. Moreover, others have reported that overexpression of NUMB in DAOY cells reduces their colony-forming ability [48]. Thus, it is tempting to speculate that knockdown of MSI2 reduces the viability of DAOY cells because of the increase in NUMB. However, further study will be needed to verify whether this is in fact the case, because MSI2 is likely to affect the expression of other important genes. In this regard, studies conducted in HEK293T cells identified >60 mRNA that associate with MSI1 [49]. Hence, MSI2 is also likely to regulate the translation of a significant number of mRNA. The reason for the reduction in the proliferation of U87 and U118 GB cells when MSI2 is knocked down is also unclear. Recently, it has been reported that elevating NUMB in U87 cells does not affect their proliferation [50]. Thus, further study will be needed to define the roles of MSI2 in brain tumor cells.

Our data also demonstrates that USP9X expression is required for the maintenance of MB and GB cells. Although the critical roles of USP9X in brain tumor cells remain to be determined, USP9X is most likely involved in the removal of ubiquitin from its target proteins [21]. In other cell types, USP9X has been reported to promote the deubiquitination of more than six proteins, including the anti-apoptotic protein MCL1 [19] and the transcription factor β-catenin [32], [33]. Recent studies have shown that the overexpression of USP9X in follicular lymphoma correlates with increases in levels of the anti-apoptotic protein MCL1 and poorer prognosis [19]. In that study, USP9X, but not a catalytically inactive form of USP9X, was shown to decrease MCL1 ubiquitination *in vitro*. USP9X has also been reported to increase the levels of β-catenin, a transcription factor linked to the growth of brain tumors [32], [33], and overexpression of USP9X has been reported to enhance the half-life and expression of β-catenin in mouse L cells and MCF7 cells, respectively [33], [34]. In addition to MCL1 and β-catenin, USP9X has been reported to deubiquitinate ITCH (an E3 ligase), MARCH7 (an E3 ligase), Smad4 (TGF-β signaling), ASK1 (oxidative stress), EFA6 (tight junction assembly) and two AMPK-related kinases (NUAK and MARK4 believed to control cell polarity and proliferation)[35], [51]–[55]. Importantly, examination of USP9X targets MCL1 and β-catenin by western blot analysis did not reveal a change in their protein levels when USP9X was knocked down in DAOY cells. Thus, future efforts will be needed to determine how knockdown of USP9X influences the fate of brain tumor cells. The impact of these studies is likely to have implications for the role of USP9X in other cancers. Recent studies have implicated USP9X in pancreatic cancer. However, USP9X may play multiple roles in pancreatic cancer. One study demonstrated that knockdown of USP9X in the human pancreatic tumor cell line, BxPC-3, reduces xenograph tumor growth, especially when combined with the BH3 mimetic ABT-737 [19]. However, in a mutant Kras mouse model, disruption of USP9X accelerated pancreatic tumorigenicity [20]. Hence, we suspect that the roles of USP9X in cancer are likely to be complex and context-dependent, similar to what has been observed for TGFβ [56].

In conclusion, we have identified >280 proteins that associate with SOX2 in DAOY MB tumor cells and demonstrate that two of the SOX2-associated proteins, MSI2 and USP9X, play key roles in the growth of MB and GB cells. Moreover, our report demonstrates that unbiased proteomic screening of proteins that associate with essential transcription factors identifies novel proteins and biological processes necessary for tumor cell growth. Hence, proteomic screens are a viable approach for identifying high-value protein candidates that warrant further study, some of which may eventually prove to be suitable therapeutic targets in the treatment of cancer.

## Supporting Information

Figure S1 **Validation of SOX2-associated proteins.** (A) Endogenous SOX2 protein was isolated from DAOY MB cell nuclear extracts using a SOX2 antibody and Protein G agarose beads. A GFP affinity antibody was used as a control. Following washes, agarose beads were boiled and the eluted protein was separated on an SDS-PAGE gel for subsequent western blot analysis. Nuclear extract from DAOY cells, used for immunoprecipitation, served as input. The blots were probed for SOX2, MSI2 and USP7. (B) An expression vector for Flag-epitope tagged SOX2 (FSOX2) was transfected into 293T cells to immunoprecipitate endogenous USP7. Twenty-four hours after transfection, nuclear proteins were isolated. Flag-SOX2 protein complexes were immunoprecipitated using M2-beads and eluted off the beads using Flag-peptide. Nuclear extract (INPUT) and immunoprecipitation eluates were used for western blot analysis, probing first for endogenous USP7 and then for Flag. (C) Expression vectors for Flag-epitope tagged MSI2 (FMSI2) and wild-type SOX2 were transfected into 293T cells. Twenty-four hours after transfection, nuclear proteins were isolated. Flag-MSI2 protein complexes were immunoprecipitated using M2-beads and eluted off the beads using Flag-peptide. Nuclear extract (INPUT) and immunoprecipitation eluates were used for western blot analysis, probing first for SOX2 and then for Flag.
(TIF)

Figure S2 **The SOX2-interactome in multiple cell types.** Description of the protein interaction landscape comparing SOX2-associated proteins identified in three different cellular contexts using the same proteomics platform. The SOX2-associating proteins in DAOY MB cells are presented in Fig. 2 and supplemental Tables S1–S3. The SOX2-interactomes in ESC and ESC undergoing differentiation (ESC-D) have been described previously ([11] and [9], respectively). A composite table listing all interacting proteins is provided in supplemental Table S6.
(TIF)

Figure S3 **Validation of MSI2 knockdown in DAOY, U87, and U118 cells.** Cells were infected with lentiviruses that express either the Scrambled shRNA sequence or the MSI2 shRNA #1 or shRNA #2 sequence. RNA was isolated from DAOY cells (A), U87 cells (B), and U118 cells (C). Expression levels of total MSI2 RNA was determined by RT-qPCR. Threshold cycle (Ct) values were calculated by normalizing all Ct values to GAPDH then subtracting the Ct value for cells infected with MSI2 shRNA #1 or shRNA #2 from the Ct value for cells infected with the Scrambled shRNA lentivirus. A negative Ct value indicates a decrease in the level of the transcript in the MSI2 knockdown cells. Multiple rounds of RT-qPCR were used to calculate an average change in Ct value, error bars represent standard error of the mean, and p values were determined by student t-test.
(TIF)

Figure S4 **Knockdown of MSI2 in U118 glioblastoma cells.** (A) Western blot analysis of MSI2 levels 96 hours after infection with Scrambled or MSI2 shRNA lentiviruses. Two isoforms were detected: isoform 1 (MSI2-1) and isoform 2 (MSI2-2). GAPDH was probed as a loading control. MSI2 levels are quantified, with levels found in the Scrambled control set to 1.00. (B) Cell growth was examined in triplicate by MTT assay 5 days after being plated at 2.5×10^4^ cells per well of a 12-well plate. The data shown are averages relative to the Scramble control. Error bars represent standard deviation and p values were determined by student t-test. P values were <.01 for both MSI2 shRNA 1. (C) Photomicrographs of U118 GB cells were taken day 6 following infection with either non-specific (Scrambled) or MSI2 targeting shRNA lentiviruses.
(TIF)

Figure S5 **Validation of USP9X knockdown in DAOY, U87, and U118 cells.** Cells were infected with lentiviruses that express either the Scrambled shRNA sequence or the USP9X shRNA #1, shRNA #2, or shRNA #3 sequence. RNA was isolated from DAOY cells (A), U87 cells (B), and U118 cells (C). Expression levels of USP9X RNA was determined by RT-qPCR. Threshold cycle (Ct) values were calculated by normalizing all Ct values to GAPDH then subtracting the Ct value for cells infected with the USP9X shRNA #1, shRNA #2, or shRNA #3 from the Ct value for cells infected with the Scrambled shRNA lentivirus. A negative Ct value indicates a decrease in the level of the transcript in the MSI2 knockdown cells. Multiple rounds of RT-qPCR were used to calculate an average change in Ct value, error bars represent standard error of the mean, and p values were determined by student t-test.
(TIF)

Figure S6 **Knockdown of USP9X in U118 GB cells.** (A) Western blot analysis to verify the knockdown of USP9X in U118 GB cells following infection with lentiviruses to introduce constitutively active shRNA against USP9X transcripts. Nuclear and cytoplasmic protein fractions were prepared 4 days after infecting cells with lentiviruses. USP9X levels are quantified, and levels in the Scrambled control are set to 1.00. (B) Cell growth was examined in triplicate by MTT assay 5 days after being plated at 2.5×10^4^ cells per well of a 12-well plate. The data shown are averages relative to the Scramble control. Error bars represent standard deviation. P values were determined by student t-test and found to be <.01 for both USP9X shRNA 1 and 2. (C) Photomicrographs of U118 GB cells following knockdown of USP9X using lentiviral delivered shRNA constructs against USP9X transcripts. On day 0, cells were infected with USP9X shRNA lentiviruses. Beginning on day 1, infected cells were selected using puromycin for 48 hours. On day 4, cells were passaged into fresh culture flasks and photographed on day 8 after infection.
(TIF)

Table S1 **SOX2-associated proteins only in Dox-treated i-SOX2-DAOY cells, P-Value (BY-adjustment)<0.05.** Proteins that were identified in all three induced M2-bead eluates, but not identified in uninduced samples, as SOX2-associated proteins by MudPIT analysis. NSAF values of the three replicates are averaged. Proteins identified were statistically significant as calculated by the BY-adjusted method (p<0.05).
(XLS)

Table S2 **SOX2-associated proteins only in Dox-treated i-SOX2-DAOY cells, p-Value (Student T-test)<0.05.** Proteins identified in 3 of 3, Dox-induced DAOY MudPIT replicates that were statistically significant according to the student’s t-test (p<0.05), but were not significant according to the BY-adjustment (p>0.05).
(XLS)

Table S3 **SOX2-associated proteins enriched in i-SOX2-DAOY.** Proteins that were identified in all three induced and at least one uninduced MudPIT sample. Enrichment values are >6-fold.
(XLS)

Table S4 **shRNA constructs used for knockdown. Lentiviral constructs were obtained from Open Biosystems.** The Open Biosystems catalogue numbers are provided.
(XLS)

Table S5 **Gene ontology classification of SOX2-associated protein identified in DAOY cells.** Gene ontologies were grouped into 14 broad categories. A yellow mark indicates that a particular protein has been associated with the indicated ontology.
(XLS)

Table S6 **Integration of SOX2-protein interactomes in multiple cell systems.** Proteins identified as SOX2-interacting proteins in DAOY MB cells (this report), U87 GB cells (unpublished data), ESC and ESC undergoing the early stages of differentiation [ESC-Diff] are listed in the left columns. Proteins common between the different interactomes are listed (progressing from Left to Right) as those found in two interactomes, three interactomes and four interactomes. The data from this table was used to construct the landscape presented in Figure 3.
(XLS)
