# Supplementary figures and images for: The SOX2-Interactome in Brain Cancer Cells Identifies the Requirement of MSI2 and USP9X for the Growth of Brain Tumor Cells
Source: PLoS One. 2013 May 7;8(5):e62857. doi: 10.1371/journal.pone.0062857 (PMC3647065; doi:10.1371/journal.pone.0062857)

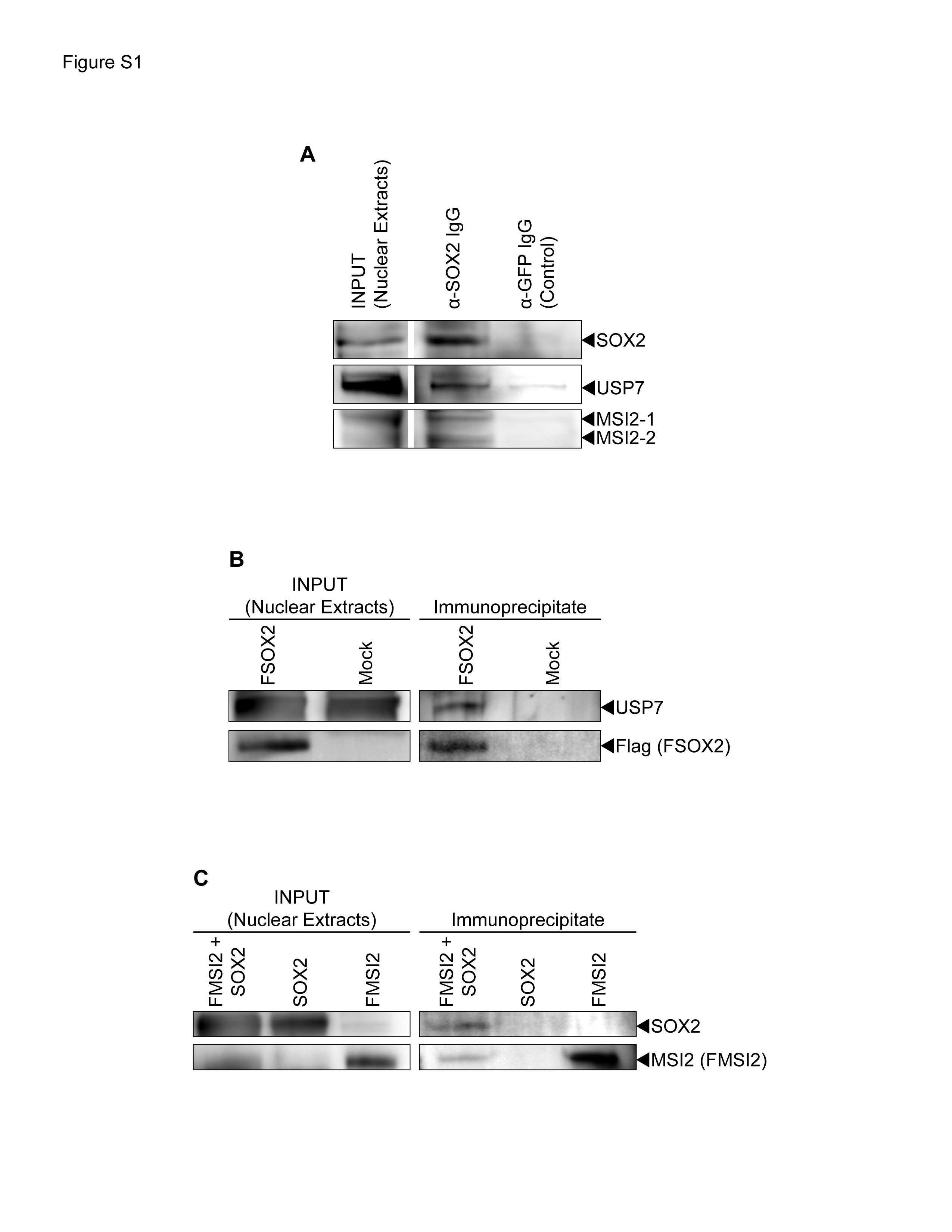

Supplement: Figure S1 — Validation of SOX2-associated proteins. (A) Endogenous SOX2 protein was isolated from DAOY MB cell nuclear extracts using a SOX2 antibody and Protein G agarose beads. A GFP affinity antibody was used as a control. Following washes, agarose beads were boiled and the eluted protein was separated on an SDS-PAGE gel for subsequent western blot analysis. Nuclear extract from DAOY cells, used for immunoprecipitation, served as input. The blots were probed for SOX2, MSI2 and USP7. (B) An expression vector for Flag-epitope tagged SOX2 (FSOX2) was transfected into 293T cells to immunoprecipitate endogenous USP7. Twenty-four hours after transfection, nuclear proteins were isolated. Flag-SOX2 protein complexes were immunoprecipitated using M2-beads and eluted off the beads using Flag-peptide. Nuclear extract (INPUT) and immunoprecipitation eluates were used for western blot analysis, probing first for endogenous USP7 and then for Flag. (C) Expression vectors for Flag-epitope tagged MSI2 (FMSI2) and wild-type SOX2 were transfected into 293T cells. Twenty-four hours after transfection, nuclear proteins were isolated. Flag-MSI2 protein complexes were immunoprecipitated using M2-beads and eluted off the beads using Flag-peptide. Nuclear extract (INPUT) and immunoprecipitation eluates were used for western blot analysis, probing first for SOX2 and then for Flag. (TIF) [file pone.0062857.s001.tif]

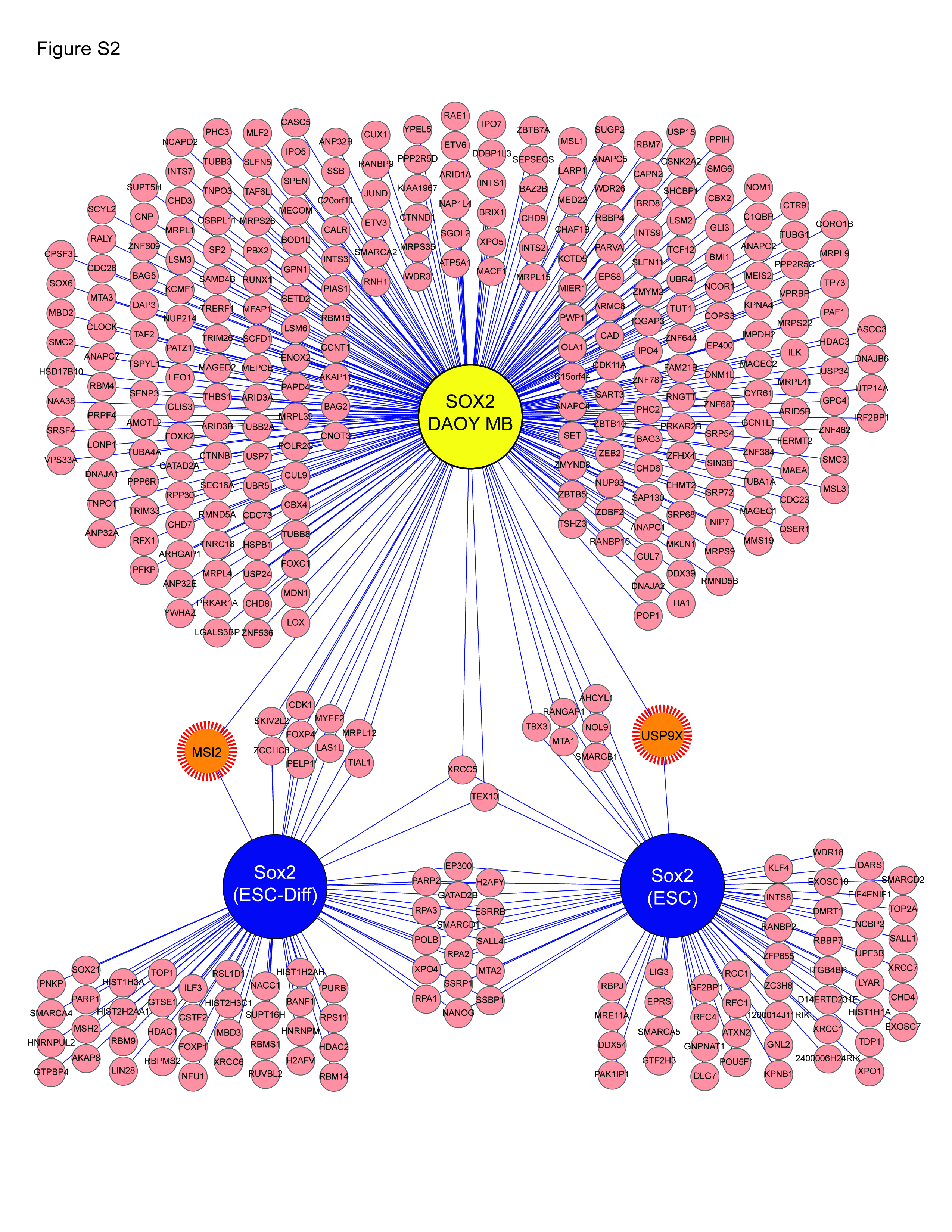

Supplement: Figure S2 — The SOX2-interactome in multiple cell types. Description of the protein interaction landscape comparing SOX2-associated proteins identified in three different cellular contexts using the same proteomics platform. The SOX2-associating proteins in DAOY MB cells are presented in Fig. 2 and supplemental Tables S1–S3. The SOX2-interactomes in ESC and ESC undergoing differentiation (ESC-D) have been described previously ([11] and [9], respectively). A composite table listing all interacting proteins is provided in supplemental Table S6. (TIF) [file pone.0062857.s002.tif]

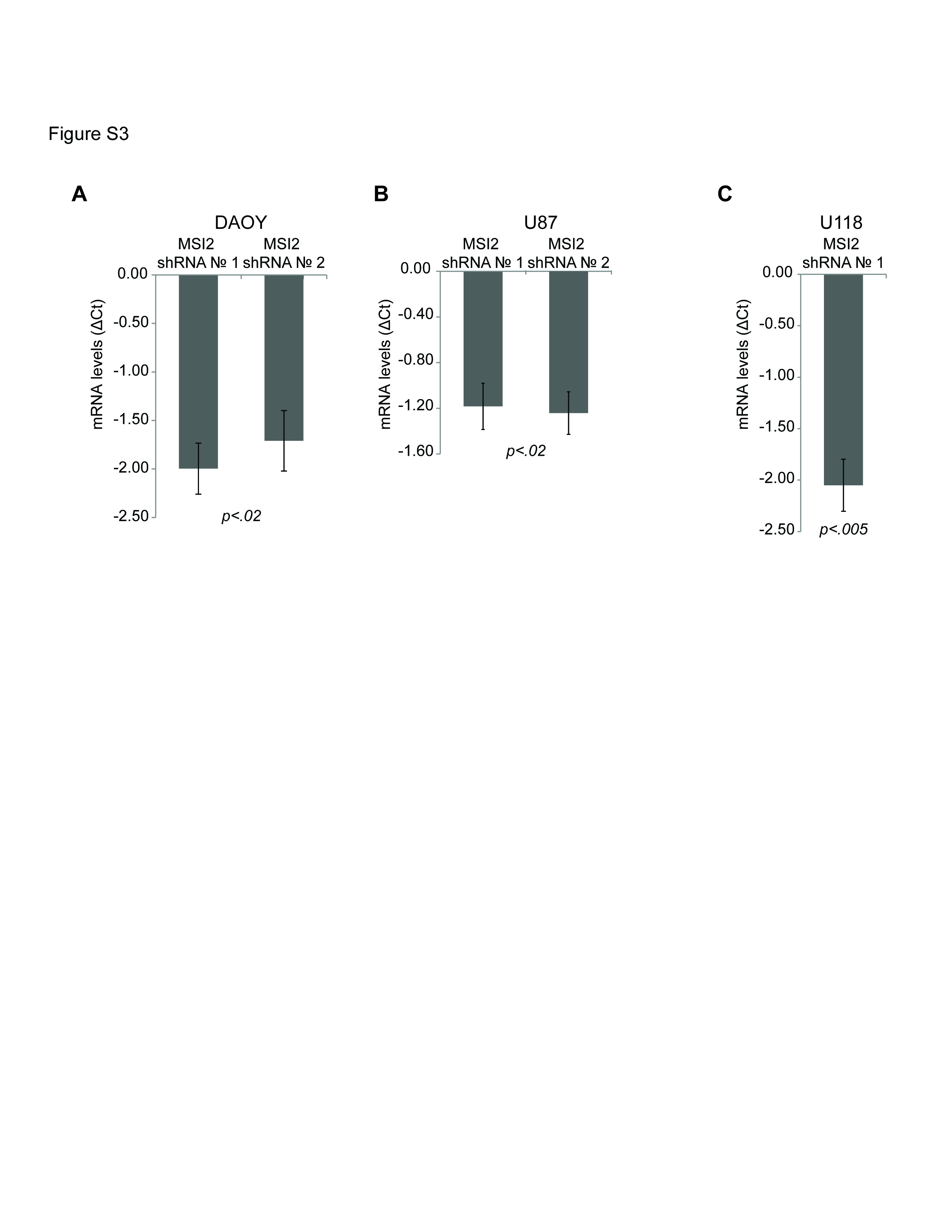

Supplement: Figure S3 — Validation of MSI2 knockdown in DAOY, U87, and U118 cells. Cells were infected with lentiviruses that express either the Scrambled shRNA sequence or the MSI2 shRNA #1 or shRNA #2 sequence. RNA was isolated from DAOY cells (A), U87 cells (B), and U118 cells (C). Expression levels of total MSI2 RNA was determined by RT-qPCR. Threshold cycle (Ct) values were calculated by normalizing all Ct values to GAPDH then subtracting the Ct value for cells infected with MSI2 shRNA #1 or shRNA #2 from the Ct value for cells infected with the Scrambled shRNA lentivirus. A negative Ct value indicates a decrease in the level of the transcript in the MSI2 knockdown cells. Multiple rounds of RT-qPCR were used to calculate an average change in Ct value, error bars represent standard error of the mean, and p values were determined by student t-test. (TIF) [file pone.0062857.s003.tif]

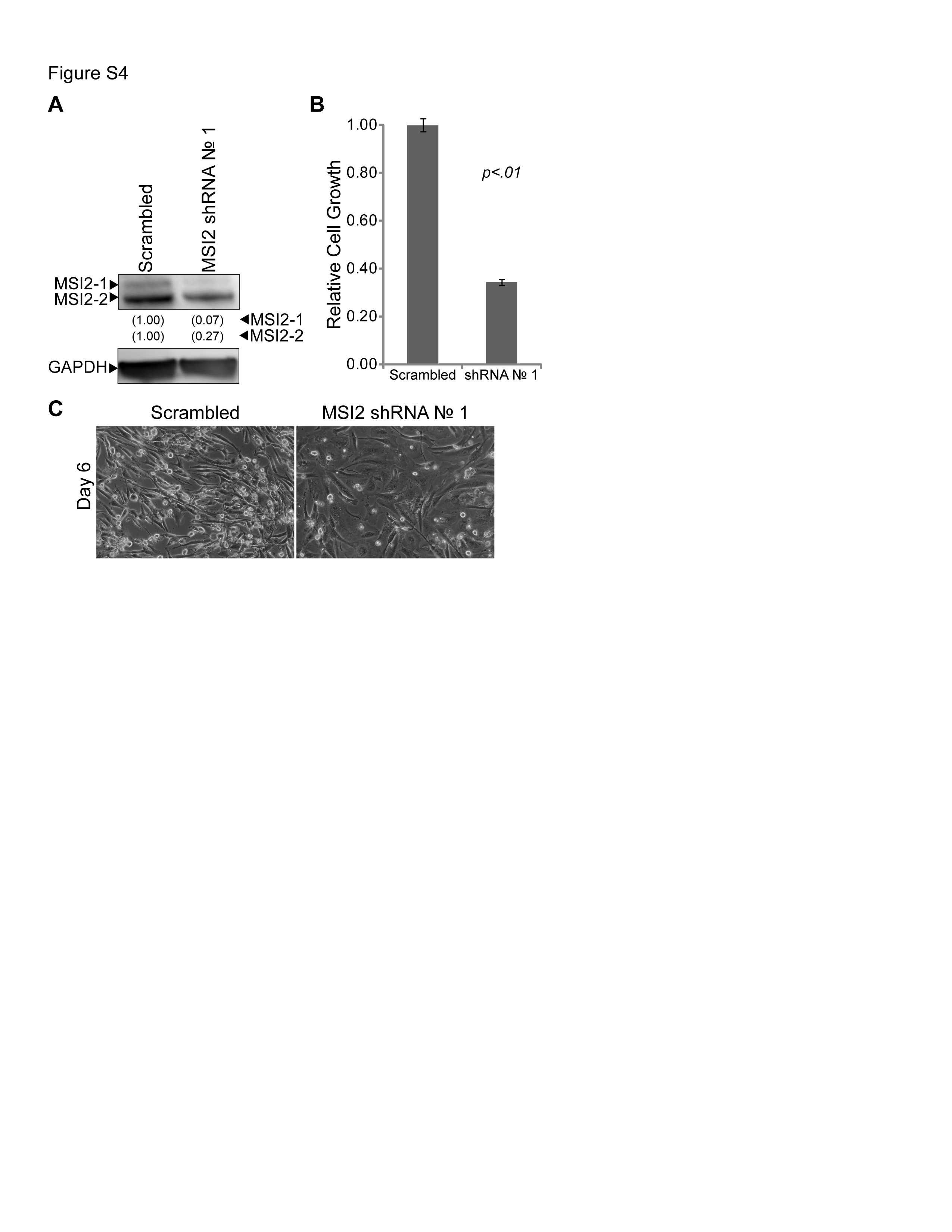

Supplement: Figure S4 — Knockdown of MSI2 in U118 glioblastoma cells. (A) Western blot analysis of MSI2 levels 96 hours after infection with Scrambled or MSI2 shRNA lentiviruses. Two isoforms were detected: isoform 1 (MSI2-1) and isoform 2 (MSI2-2). GAPDH was probed as a loading control. MSI2 levels are quantified, with levels found in the Scrambled control set to 1.00. (B) Cell growth was examined in triplicate by MTT assay 5 days after being plated at 2.5×10^4^ cells per well of a 12-well plate. The data shown are averages relative to the Scramble control. Error bars represent standard deviation and p values were determined by student t-test. P values were <.01 for both MSI2 shRNA 1. (C) Photomicrographs of U118 GB cells were taken day 6 following infection with either non-specific (Scrambled) or MSI2 targeting shRNA lentiviruses. (TIF) [file pone.0062857.s004.tif]

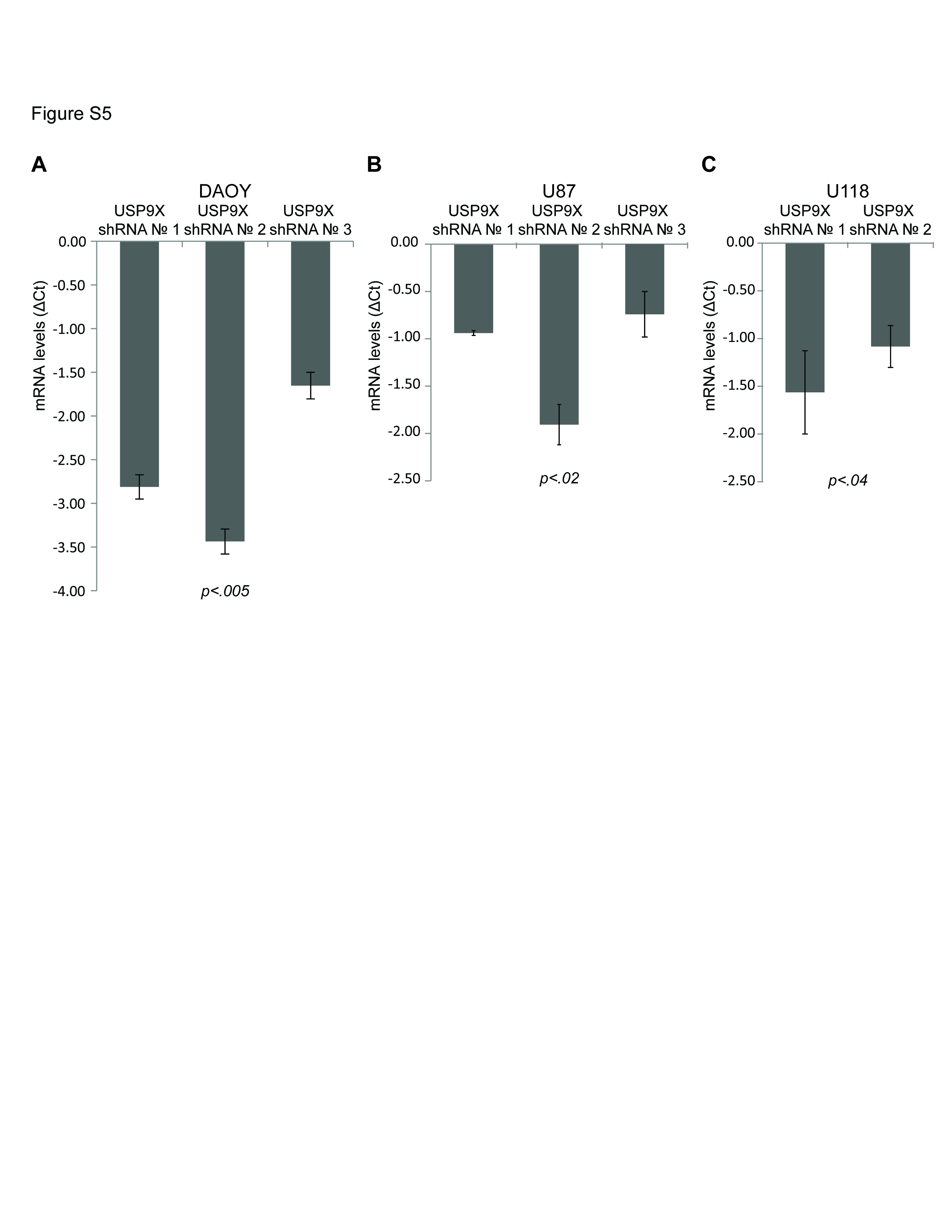

Supplement: Figure S5 — Validation of USP9X knockdown in DAOY, U87, and U118 cells. Cells were infected with lentiviruses that express either the Scrambled shRNA sequence or the USP9X shRNA #1, shRNA #2, or shRNA #3 sequence. RNA was isolated from DAOY cells (A), U87 cells (B), and U118 cells (C). Expression levels of USP9X RNA was determined by RT-qPCR. Threshold cycle (Ct) values were calculated by normalizing all Ct values to GAPDH then subtracting the Ct value for cells infected with the USP9X shRNA #1, shRNA #2, or shRNA #3 from the Ct value for cells infected with the Scrambled shRNA lentivirus. A negative Ct value indicates a decrease in the level of the transcript in the MSI2 knockdown cells. Multiple rounds of RT-qPCR were used to calculate an average change in Ct value, error bars represent standard error of the mean, and p values were determined by student t-test. (TIF) [file pone.0062857.s005.tif]

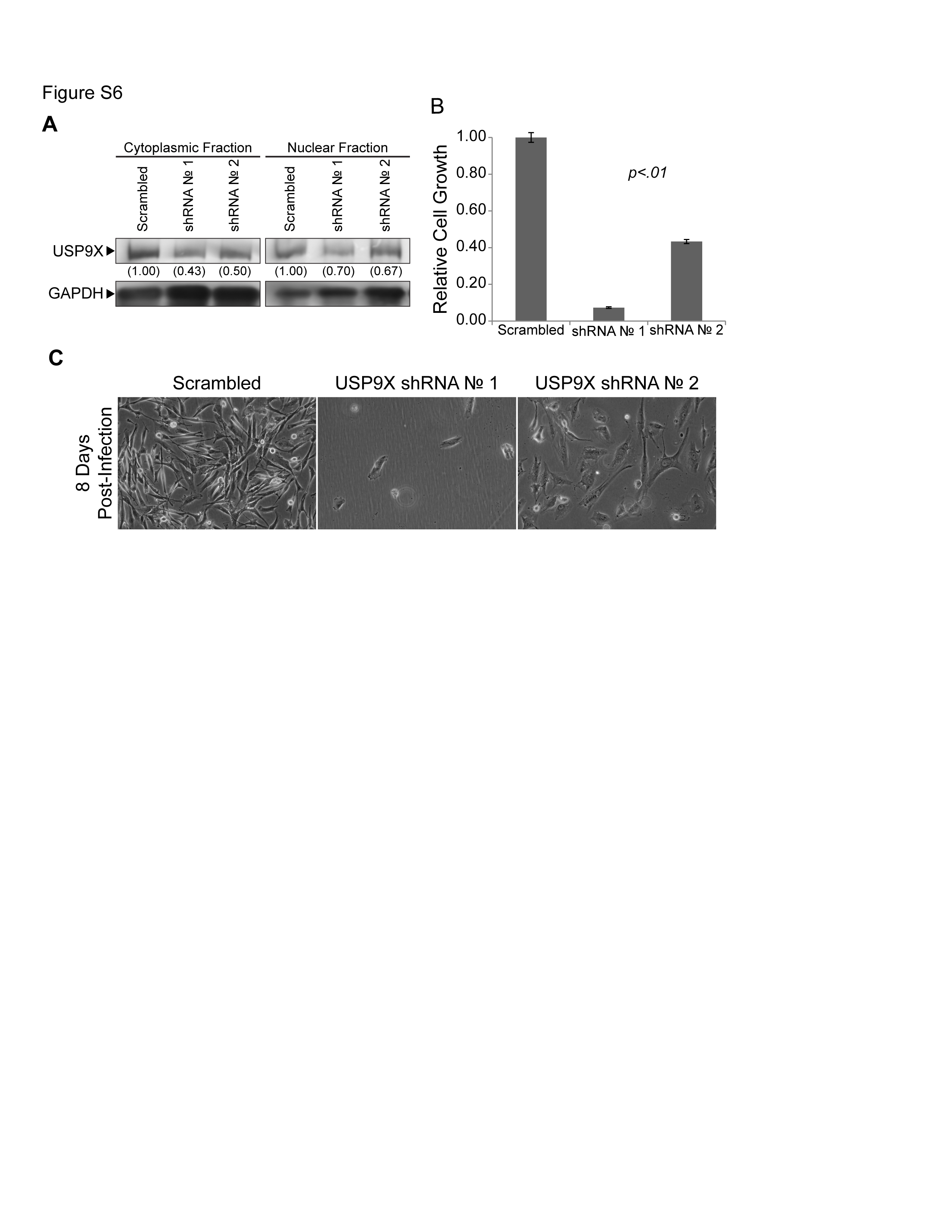

Supplement: Figure S6 — Knockdown of USP9X in U118 GB cells. (A) Western blot analysis to verify the knockdown of USP9X in U118 GB cells following infection with lentiviruses to introduce constitutively active shRNA against USP9X transcripts. Nuclear and cytoplasmic protein fractions were prepared 4 days after infecting cells with lentiviruses. USP9X levels are quantified, and levels in the Scrambled control are set to 1.00. (B) Cell growth was examined in triplicate by MTT assay 5 days after being plated at 2.5×10^4^ cells per well of a 12-well plate. The data shown are averages relative to the Scramble control. Error bars represent standard deviation. P values were determined by student t-test and found to be <.01 for both USP9X shRNA 1 and 2. (C) Photomicrographs of U118 GB cells following knockdown of USP9X using lentiviral delivered shRNA constructs against USP9X transcripts. On day 0, cells were infected with USP9X shRNA lentiviruses. Beginning on day 1, infected cells were selected using puromycin for 48 hours. On day 4, cells were passaged into fresh culture flasks and photographed on day 8 after infection. (TIF) [file pone.0062857.s006.tif]
